# Birth attendants’ hand hygiene compliance in healthcare facilities in low and middle-income countries: a systematic review

**DOI:** 10.1186/s12913-020-05925-9

**Published:** 2020-12-03

**Authors:** Giorgia Gon, Mícheál de Barra, Lucia Dansero, Stephen Nash, Oona M. R. Campbell

**Affiliations:** 1 London School of Hygiene and Tropical Medicine, Keppel Street, London, WC1E 7HT UK; 2 Brunel University London, Department of Life Sciences, Uxbridge, UK; 3 Independent researcher, Turin, Italy

**Keywords:** Hand hygiene, Maternal and newborn health, Labour, Healthcare workers

## Abstract

**Background:**

With an increasing number of women delivering in healthcare facilities in Low and Middle Income Countries (LMICs), healthcare workers’ hand hygiene compliance on labour wards is pivotal to preventing infections. Currently there are no estimates of how often birth attendants comply with hand hygiene, or of the factors influencing compliance in healthcare facilities in LMICs.

**Methods:**

We conducted a systematic review to investigate the a) level of compliance, b) determinants of compliance and c) interventions to improve hand hygiene during labour and delivery among birth attendants in healthcare facilities of LMICs. We also aimed to assess the quality of the included studies and to report the intra-cluster correlation for studies conducted in multiple facilities.

**Results:**

We obtained 797 results across four databases and reviewed 71 full texts. Of these, fifteen met our inclusion criteria. Overall, the quality of the included studies was particularly compromised by poorly described sampling methods and definitions. Hand hygiene compliance varied substantially across studies from 0 to 100%; however, the heterogeneity in definitions of hand hygiene did not allow us to combine or compare these meaningfully. The five studies with larger sample sizes and clearer definitions estimated compliance before aseptic procedures opportunities, to be low (range: 1–38%). Three studies described two multi-component interventions, both were shown to be feasible.

**Conclusions:**

Hand hygiene compliance was low for studies with larger sample sizes and clear definitions. This poses a substantial challenge to infection prevention during birth in LMICs facilities. We also found that the quality of many studies was suboptimal. Future studies of hand hygiene compliance on the labour ward should be designed with better sampling frames, assess inter-observer agreement, use measures to improve the quality of data collection, and report their hand hygiene definitions clearly.

## Background

Globally, infection contributes to at least 9% of maternal deaths [1] and 16% of neonatal deaths [2], the vast majority of this burden concentrates in low and middle income countries (LMICs). Hand hygiene during birth has been long recognised as a key infection prevention opportunity [3, 4]. With an increasing number of women delivering in healthcare facilities in LMICs [5], appropriate hand hygiene compliance of healthcare workers on the labour wards is pivotal to preventing infections.

Several systematic reviews have been published on the compliance, determinants and interventions to improve healthcare workers hand hygiene across the facility environment [6–10]; only two of these reviews include studies from low resource healthcare facilities, none of which provide estimates for the labour ward [7, 8]. Erasmus et al. report a median hand hygiene compliance of 40% for studies from high-income countries [6]; the other, more recent, reviews focus on evaluating existing interventions and do not report summary estimates of compliance, but there is value in collating estimates from observational studies too.

Currently there are no estimates of how often birth attendants comply with hand hygiene, or of the factors influencing their compliance in healthcare facilities in LMICs. Hand hygiene compliance in LMICs may differ in levels and determinants compared to those in high-income countries (HICs), where most published evidence is. For example, there are cultural and contextual elements around the process of labour and delivery that might influence hand hygiene compliance of healthcare workers such as unpredictable workloads, unreliable water supplies, or the concept of pollution and purity around delivery – important among healthcare workers in India and Bangladesh [11, 12]. Finally, detailed estimates on compliance in LMICs and their determinants are useful to inform whether interventions are needed, and how to tailor them.

The aim of this paper is to systematically review the literature from LMICs to:

1. Estimate birth attendants’ hand hygiene compliance during labour and delivery in healthcare facilities
2. Assess the quality of the studies reporting these estimates
3. Investigate what factors influence hand hygiene compliance
4. Estimate the effectiveness of interventions aimed at increasing hand hygiene compliance
5. Estimate intra-cluster correlation for hand hygiene compliance comparing variation within and between facilities

## Methods

The search was conducted on the 1^st^ of September 2020, updating earlier searches on the 24th of April 2018 and on the 27th of January 2016 over EMBASE, MEDLINE, CINHAL, and the WHO regional databases (the website we used for the latter was not accessible during the last search in spite several attempts). We used a comprehensive set of search terms based on previous systematic reviews [8, 13, 14] and consulted the London School of Hygiene and Tropical Medicine librarian. The search themes included hand hygiene and maternity ward terms with international spelling variations, and it was restricted to LMICs. Additional file 1 details the strategy. Peer reviewed articles were eligible for inclusion, while abstracts and conference proceeding were not. All texts were reviewed using Endnote X7. No protocol was registered for this review.

Duplicates were removed, and titles and abstracts screened for any mention of hand hygiene compliance in labour wards. Two reviewers independently applied the inclusion criteria to the selected full texts. Any discrepancy was resolved through discussion. Once full texts were selected, one author screened references to search for other relevant studies that might be eligible for inclusion. The inclusion criteria were:

- Studies with either of the following estimates for the specific group of healthcare workers attending labour and delivery or working on the labour ward:
  - A measure of frequency for hand hygiene compliance (observed or other objective method; self-reports were not included)
  - OR an effect size (odds ratio, rate ratio, risk ratio) of factors driving hand hygiene (observed or other objective method; self-reports were not included)
- LMICs based studies
- Peer-reviewed studies
- Intervention or observational studies
- Quantitative studies
- Studies in any language

Data extraction was done by one author and checked by another. The data extraction form included study type, intervention details, country, urban-rural location, type of healthcare facility, staff cadre, facility ward specification, availability of hand hygiene infrastructure (soap, water, handrub), sample size, sample selection, analysis methods, measurement tools, and the effect size of hand hygiene determinants. We extracted the estimates of hand hygiene compliance by healthcare workers before aseptic procedures (or compliance estimates which were likely to include before aseptic procedure opportunities) for a) types of patient-attendant interactions that could occur during labour and delivery, or b) healthcare workers working in the labour ward. We specifically focused on estimates reflecting hand hygiene opportunities before aseptic procedures these because these are the most pivotal to infection prevention. For each estimate we extracted the hand hygiene definition, the numerator, denominator, the percentage compliance estimates, the number of staff or women observed, the staff cadre, the number of facilities, and the intervention stage details underpinning the individual estimate. We calculated the percentage compliance for each included study where this was possible. We contacted the corresponding author (or if this was not published, the first or senior author whose email we found via their department or on *researchgate)* when it was not clear from the paper whether a) their observation included procedures around labour and vaginal delivery; or b) when the hand hygiene definition was unclear and the tool used was not available.

Key measures of bias and quality were included in the data extraction. For randomised controlled trials we intended to use the CONSORT guidelines to assess quality. For observational studies, we assessed quality using checklist we developed using eight items adapted from the STROBE guidelines’ [15] methods section (as recommended by Sanderson and colleagues) [16], to the specific context of observing hand hygiene in healthcare settings. Items included assessing 1) sampling methods, 2) quality of data collection, 3) description of the data collectors background, 4) whether inter-observer agreement was estimated, 5) the definition of hand hygiene compliance, 6) details of the tool used for observation, 7) whether study aims were concealed from the study participants and 8) whether the statistical procedures were described. Items were scored *positively* or *negatively*, except for items 1, 3 and 6 where we added an extra option of *partially met* when only one of two criteria was met, and item 7 which could also be scored as *unclear*.

Intra-cluster correlation (ICC) accounts for the relatedness of data by comparing the variance within clusters with the variance between clusters; it is useful for designing and analysing observational and intervention studies. To obtain the ICC for hand hygiene compliance of the included studies comparing the variation in compliance between and within facilities, we also contacted the authors of studies with multiple facilities (clusters) to ask for:

- Either, the following single measures:
  - The standard deviation exhibiting how the cluster means vary from the population mean from cluster to cluster σ_*b*_ (between-cluster variation)
  - The standard deviation exhibiting how individual values vary from their cluster mean from individual to individual σ_*w*_ (within-cluster variation). Individuals are birth attendants in our review.
- Or, the overall estimated ICC (ρ) = ρ = σ_*b*_^2^ / (σ_*b*_^2^+ σ_*w*_^2^)

We aimed to conduct pooled analysis of the estimates by hand hygiene compliance estimated using similar outcome definitions, measurement tools or investigating similar interventions, unless there are differences in setting or risk of bias; where studies did not use similar outcomes, measurement tools or investigate similar interventions, estimates were described.

We followed the PRISMA guidelines for systematic reviews to report our methods and findings (see Additional file 2) [17].

## Results

**Fig. 1:**
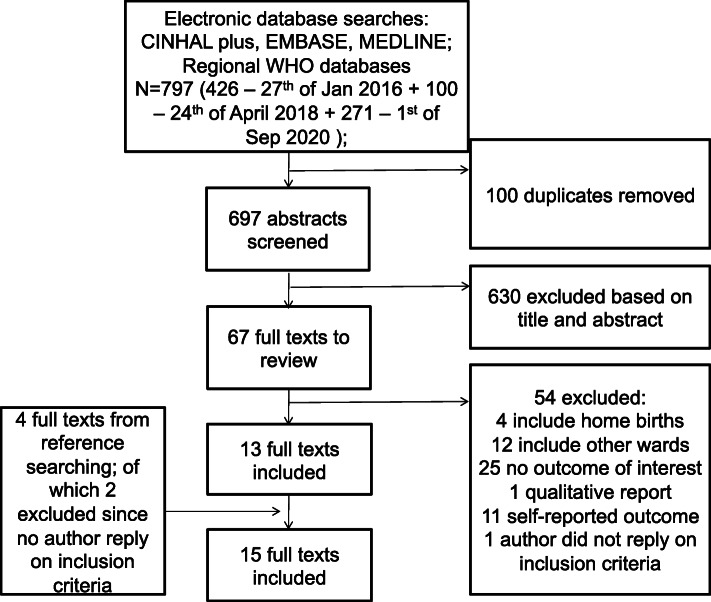
Systematic search flow diagram

After removing duplicates (100), we obtained 697 results across the four databases and reviewed 71 full texts of which 4 are from reference searching (Fig. 1). We ultimately included fifteen that met our inclusion criteria. The reasons for excluding the fifty-seven studies are in Fig. 1, with the most common being that the study did not report on the outcome of interest, i.e. hand hygiene of healthcare workers during labour or delivery, or in the labour ward. In two articles which were identified via reference searching, it was unclear whether labour and delivery were being studied, and the author of the paper did not reply to enquiry, so these papers were not included.

Of the fifteen included studies, seven were in Sub-Saharan Africa (Zanzibar-Tanzania, Zimbabwe, two in Ghana, and three in Nigeria), two were in Iran, the rest were located in in South-East Asia: three in India, one in Vietnam, one in the Thai-Myammar border, and one spanned several countries (Cambodia, Lao People’s Democratic Republic, Mongolia, Papua New Guinea, Philippines, Solomon Island, and Vietnam) – see Table 1. The studies were published between 1993 and 2020, with only one study being published prior to 2008. Four studies were conducted in a single facility. Six of the nine studies did not report any information on hand hygiene infrastructure (Table 1); one study discussed how inconvenient the sink location was; one study selected the hospital based on it generally having supplies to provide good quality of maternal care; three studies reported on the general availability of supplies (two positively and one negatively), but it is unclear what elements of hand hygiene infrastructure were surveyed if any. Only four studies reported specifically on the availability of hand hygiene infrastructure. Two of these studies reported that needed supplies were present, except for handrub in the first study [32], and disposable towel in the second [19]; one reported that not all the facilities had needed supplies, but the percentage refers to a wider set of facilities compared to the one observed for hand hygiene [27]; and one reported the availability of 24-h running water (52% of facilities) and soap (65% of facilities) (Table 1) [24].

**Table 1:** Study characteristics

	Asp (2011) [18]	Buxton (2019) [19]	Changaee (2014) [20]	Cronin (1993) [21]	Danda (2015) [22]	Delaney (2017) [23]	Friday (2012) [24]	Gon (2018) [25]	Hoogenboom (2015) [26]	Mannava (2019) [27]	Phan (2018) [28]	Simbar (2008) [29]	Spector (2012) [30]	Tyagi (2018) [31]	Yawson (2013) [32]
**Country; site**	Nigeria; Lagos	Nigeria; Ebonyi and Kogi	Iran; Lorestan	Ghana; North & South Birim Districts	Zimbabwe	India; Uttar Pradesh	Nigeria; Edo State	Zanzibar, Tanzania	Thai-Myanmar border; Mae La refugee camp	Cambodia, Lao PDR, Mongolia, Papua New Guinea, Philippines, Solomon Islands, Viet Nam	Vietnam; Ho Chi Minh City	Iran; Kurdistan	India; Karnataka	India; Telangana, Andhra Pradesh	Ghana; Accra
**Study design**	Cross-sectional	Cross-sectional	Cross-sectional	Cross-sectional	Cross-sectional	Repeated Cross-sectional (nested in randomised trial)	Cross-sectional	Cross-sectional	Cross-sectional	Cross-sectional	Pre-post multi-component intervention	Cross-sectional	Pre-post multi-component intervention	Cross-sectional	Cross-sectional
**Facility type**	1 secondary and 1 tertiary maternity care facility	2 Primary healthcare facilities 2 secondary healthcare facilities 2 Tertiary healthcare facilities	9 public hospitals	1 public hospital, 6 public health posts, 5 private maternity homes	2 University of Zimbabwe Central Hospitals i.e. National referral hospitals	15 healthcare facilities of the 60 selected for the intervention. The 60 facilities varied between primary and community health centres and first level referral units.	63 healthcare facilities including primary health centers, private clinics, two secondary/district hospital, 2 tertiary/teaching hospitals	1 referral hospital 1 maternity Hospital 3 Cottage Hospitals 1 private Hospital 3 district Hospitals 1 Primary healthcare Unit	Shoklo Malaria Research Unit Clinic	76 first level referred hospital 25 tertiary hospitals	Hung Vuong University Hospital	Be-Sat Hospital of Sanandaj and Hafte-Teer Hospital of Beejar	Sub-district level hospital (basic emergency obstetric care and C-sections)	26 Public secondary healthcare facilities 4 public tertiary healthcare facilities 5 private tertiary healthcare facilities	Korle-Bu Teaching Hospital (tertiary healthcare facility)
**Unit/ward**	Maternity ward	Labour ward	Unclear. Presumably labour ward	Unclear. Presumably labour ward	Labour & postnatal ward	Labour wards	Delivery wards	Labour wards	Birth centre	Delivery rooms	Delivery suite	Labour & delivery wards	Unclear. Presumably labour ward	Labour ward	Emergency Room and Labour ward
**Effect size**	None	None	None	None	None	None	None	None	None	None	None	None	None	None	None
**Intervention**	None	None	None	None	None	Introduction of Safe Childbirth Checklist with peer coaching	None	None	None	None	Yes; educational intervention	None	Yes; testing checklist	The study is part of a baseline evaluation of a quality improvem. Intervention	None
**Health professionals involved**	Midwives	Doctors, midwives, auxiliary staff	Unclear. Midwives are mentioned in the discussion	Midwives, midwives’ assistants and lay women trained by midwives	Midwives	Any birth attendants	Attending midwives	All staff involved in assisting deliveries	Literate skilled birth attendants resident in the camp and trained by the clinic (not previously trained in midwifery)	Unclear. Any birth attendants	All healthcare workers in the delivery suite. Across all departments in the study they capture doctors, nurses, midwives and technicians^a^	Unclear	Any healthcare worker^a^ (nurses & obstetricians) who cared for women and newborns from admission for childbirth to discharge	Health care providers working in the labour ward	Doctors and nurses
**Type of patient-attendant interactions**	52 women during delivery and immediate postpartum	31 women in active labour (cervical dilation> 3) and admitted to delivery	200 (low risk) pregnant women^b^	18 vaginal deliveries and 22 neonatal cord-care events	20 observations in the labour and 17 in the postnatal wards	1277 deliveries. Specifically before pushing and soon after birth.	Unclear. Mentions examination and procedures requiring gloves	781 aseptic procedures during labour and delivery	20 births	371 deliveries	All types of hand hygiene opportunity in the delivery suite	96 women with low risk pregnancies^b^	405 vaginal examinations at admission and 388 deliveries	242 pre-vaginal examination and 235 deliveries	Unclear
**Observation period**	May 2010	4 weeks in July 2017	Unclear	2 Months. August–September 1991	May to June 2014	6 to 12 weeks during the intervention from Dec 2014 to Sep 2016	January to May 2011	November 2015–April 2017	6 weeks. Nov-Dec 2008	Unclear 2016–2017	August 2014–May 2015	Throughout 2006	Baseline: Jul-Sept 2010; Endline: Sept.-Dec 2010	May 2016–August 2016	3 weeks. September 2011
**Data collectors**	Unclear	Qualified midwifes	Unclear	Project director and co-director (a Ghanaian nurse)	3 midwives researchers. 2 working at the study institution. 1 just left the study institution	Trained nurses	Unclear. Trained staff	3 Trained midwifes for each labour ward	2 Dutch midwifery students (4th year)	Trained doctors, nurses, midwives or public health professionals	6 infection control staff trained in direct observation. Unclear if worked in study institute	Unclear. “Researcher”	Student nurses previously unknown to hospital staff with no clinical responsibility	12 nursing graduates trained for direct observation	6 nurses specifically trained in infection control
**Tool used for observation**	Checklist developed for study. Based on protocol by Christensson et al. (2001) [33]	Standardised direct observation tool developed for study. Based on a previous tool developed for a qualitative care study. But it references a report [34]; unclear	Checklist developed for study. Content validity assessed	Checklist created for study using criteria from e.g. the WHO Global Programme on AIDS, 1989	Checklist for labour ward developed for study	The WHO Safe Childbirth Checklist	Adapted tool from a previous study based in India. Unclear reference	Observation tool developed for study based on WHO guidelines on hand hygiene in healthcare, 2009 and WHO hand hygiene technical reference manual, 2009	Checklist developed for study, drawing on WHO Safe Motherhood Needs Assessment v1.1 2001	﻿Standard checklist based on EENC Module 1: Annual Implementation Review and Planning Guide	Checklist using the WHO Guidelines on Hand Hygiene in Health Care, 2009. Observation checklist content validity reviewed by MoH and University staff	Tool developed for study based mainly on WHO’s protocol of normal birth, 1997& 2006	WHO Safe Childbirth Checklist presumed to have been used	﻿Checklist adopted from the WHO concept of five moments of hand hygiene, 2010	Modified version of the WHO form for hand hygiene direct observation, 2010
**Study aim disclosed to participants**	Unclear. Non participant observation	Study aims were explained to the participants and to the whole staff	Unclear	Participants not told when observation would take place or what practices were observed	Observers were “inside participants” assisting midwives in their work. Checklist was filled after procedures in private	Birth attendants were aware of the observation –observation included many aspects of quality of care; not hand hygiene only.	Unclear. Birth attendants were not previously informed of the walk-in visits but they were not blinded.	Healthcare workers were aware of the observation but were told that the observation was about overall quality of care (not specifically hand hygiene)	Unclear	Unclear	Unclear. Healthcare workers were aware of the observation period	Study aims were explained to the participants midwives	Nature of the intervention, which included awareness practices included in the WHO Safe Birth Checklist (e.g. hand hygiene), presumably clear to participants	Unclear	Unclear. Health workers in these service centres were not aware of being observed
**Sampling**	Unclear	The facilities with the highest number of deliveries were selected for each state (one primary, one secondary and one tertiary). Any woman who met inclusion criteria and gave consent was invited to participate up to 5 woman per facility. Unclear how the timing of facility visits was scheduled	Non-random quota sampling used to recruit 200 women. 10–30 selected in different stages of labour in each hospital. Sample size calculations justified. Unclear how different stages of labour or women and timing of visits were selected	Observation took place when the project staff visited a facility at a time when a woman was in labour. All midwives on duty when observation took place were included. Occasionally called by facility when delivery expected. Not clear how the timing of facility visits for observation were scheduled	All midwives at the time of the observation were included in the study. Not clear how the timing for facility visits were scheduled	15 facilities for independent observation were based on pragmatic sample. Independent observer visited facilities during non-intervention days for a period of 6 to 12 weeks with the goal to reach 240 pose points in each facility. A mother was observed for as many pause points as possible. Not clear how the timing for facility visits were scheduled	Public and private health facilities with high caseloads of pregnant women were selected from eight Local government areas. Presumably one walk-in visit for each hospital. Not clear how visits were scheduled and how many deliveries were observed	Observation occured in the 10 highest-volume labour wards for a mode of 6 days each (5–14 days range) for 24 h a day. All attendants. Involved in assisting deliveries during observation. Not very clear how they selected which healthcare worker to observe. Sample size calculations justified.	Unclear	Random selection of 3 national or regional tertiary hospital, 4–12 provincial hospital and 2–4 district hospitals in each country. Deliveries were observed over 1–2 days in each hospital selected. Un clear the selection of delivery observation period; but it mentioned observation was limited to the time of the assessments. Not clear how the timing for facility visits were scheduled	Unclear	Women’s selection – quota sampling (1 in 3 women) proportionally divided between morning, evening and night shifts. Not clear if all women received the full set of observations	Observation took place 24-h for a minimum of 6 days weekly; unobserved days were random. Observation was carried out at admission, from start of pushing to 1 h after birth, discharge. Unclear how women were selected each stage	In the labour room observer spent 6 h a day (either morning or evening shift) for 6 days observing 2–3 mothers a day. No details on how visits or shifts were scheduled. Only one woman was observed at the time	Observation in times & locations with high care density. Each centre was observed at a different time of day for 2 days between 8 AM-5 PM. Not clear how they selected which healthcare worker to observe
**Water/Soap/handrub availability**	Unclear. Sinks were not located in convenient locations	All facilities had soap and water in the delivery unit but during 1 observation there was no soap. All delivery units had a sink with connected tap available but 2 used veronica buckets. No disposable towerls	None	Unclear. Only reported missing items. Water, soap, handrub were not mentioned as missing	Unclear. They report broadly that basic supplies were often unavailable (not clear is specific to hand hygiene supplies)	Unclear	24-h running water was present in 52% of the observed facilities. Soap in 65% of facilities.	Unclear	Unclear. All essential equipment for standard antenatal care, and essential care of obstetric complications was present.	147 hospitals assessed for WASH services. 72% of hospitals had clean sinks with running water, soap or handrub in the delivery rooms. Data is not specific for the 101 hospital where deliveries were observed	None	None	Unclear. Hospitals selected based on general availability of supplies	unclear	Resources observed once. Water, soap and single-use towel for drying available on labour ward. Handrub not available

^a^Unclear if all mentioned cadres were observed during labour and delivery

^b^Unclear whether hand hygiene was observed for all of these

### Quality of primary studies

**Fig. 2:**
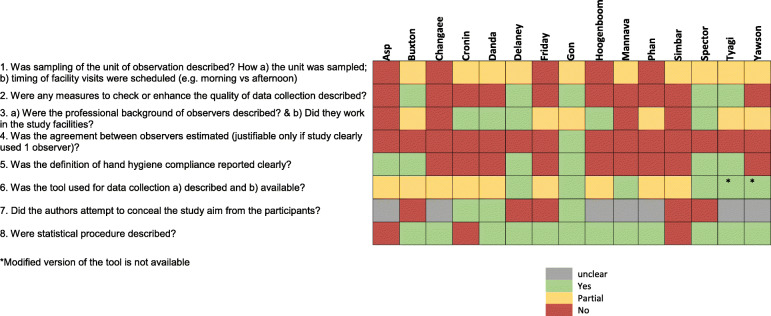
Risk of bias and quality assessment

All studies used observation as their primary method of data collection. The methods were described in most articles only partially. The lowest ranked quality indicators were 1) sampling, 2) methods to enhance data quality during data collection, 3) measurement of inter-observer agreement, and 4) the level of description of the hand hygiene compliance definition – see Fig. 2.

#### Sampling

We required two aspects of the sampling methods to be described: a) how the unit of observation (e.g. woman, procedure or healthcare worker) was sampled and b) how the timing of facility visits were scheduled. None described both aspects sufficiently; five articles did not describe them at all. As detailed in Table 1, it was often unclear how different women or healthcare workers were selected for observation.

#### Quality during data collection

Only four articles directly addressed the procedures adopted to ensure a better quality of data collection. Buxton et al. report that data collection did not start until results were consistent during the training period [19]. Spector et al. included on-site reviews of all observation forms within 72 h by the local study coordinator, and in-built data management checks confirming the data collected were logical [30]. Gon et al. provided tailored feedback to data collectors based on the results of the inter-observer exercise run in the first month [25]. Tyagi et al. incorporated quality checks in their tool as a results of the training [31].

#### Inter-observer agreement

Gon et al. is the only study that reports the results of interobserver agreement. This was calculated between pairs of data collectors in the first month of the study; the range of kappa statistics results was 0.73–0.93 for three pairs of data collectors [25]. Buxton et al. report that inter-rater reliability was monitored during the training period but do not report their results [19]. Spector et al. [30] attempted to examine agreement between observers – specifically, they reported that periodic assessments were used to confirm that data collectors achieved 100% concordance on a sample of three observations. Yawson and Hesse only report that different pairs of technical personnel visited the unit each day in order to limit intra-observer bias [32].

#### Definition of outcome

Hand hygiene compliance was not defined clearly in most studies. Each definition is reported in detail in Table 2. Some studies did not report whether soap use or handrub was necessary to achieve adequate hand hygiene and did not refer to guidelines that specifically do [20, 22, 24, 26, 27, 29]; in addition often studies did not report if other aspects of hand hygiene such as the sequence of actions preceding or following hand washing/rubbing, technique or duration were assessed in the summative compliance estimates – except for Gon et al. and Buxton et al. We describe here the studies where definitions presented additional anomalies. Yawson and Hesse, and Phan et al. mentioned that they followed the hand hygiene guidelines by the WHO but it was not clear which aspects of the guidelines they included. Buxton et al. also mentioned that they followed the WHO guidelines but created their own categories of hand hygiene ranging from the least hygienic (category 5) to the most hygienic (category 1) which included hand washing with soap, new gloves applied and no potential recontamination. Cronin et al., Danda et al., Friday et al. and Hoogenboom et al. chose a less informative definition of hand hygiene compliance because their denominator referred to whole individuals, group of individuals or facilities rather than specific patient-healthcare worker interactions (e.g. *hand washed at least once* or *at least one birth attendant washed hands*). In Changaee et al., it was not clear how they calculated their estimate of *desirable* hand washing.

**Table 2:** Compliance estimates before aseptic procedures during labour or delivery

	Asp (2011) [18]	Buxton (2019) [19]	Changaee (2014) [20]	Cronin (1993) [21]	Danda (2015) [22]	Delaney (2017) [23]	Friday (2012) [24]	Gon (2018) [25]	Hoogenboom (2015) [26]	Mannava (2019) [27]	Phan (2018) [28]	Simbar (2008) [29]	Spector (2012) [30]	Tyagi (2018) [31]	Yawson (2013) [32]
**1st Estimate**															
**Outcome definition**	Hand washing with soap or hand disinfection	Hand hygiene compliance before aseptic procedures. Hand washing with soap and new gloves applied without potential recontamination^c^.	Unclear. Desirable hand washing. Estimated % was compliance with desirable status defined as 68–100% score. Unclear if soap necessary	Number of midwives who hand scrubbed with Dettol or soap and water	Whether each midwife washed her hands at least once. Unclear if with soap	Hand hygiene compliance measured by independent observers during non-intervention days. Hand washing with soap and water or alcohol rub	Unclear. Hand hygiene compliance at the facility level. Unclear if soap necessary and if handrub allowed.	Hand hygiene compliance before aseptic procedures. Steps included hand washing ﻿with alcohol-based hand rub or wash hands with soap and water, avoided recontamination and donning gloves^c^.	Hand washing of at least one of the birth attendant present. Unclear if with soap	Hand hygiene compliance. Unclear if with soap	Hand hygiene compliance is the ratio of the number of performed actions to the number of opportunities. Presumably, soap & water or handrub necessary^c^.	Hand washing; Unclear if with soap	Hands washed with clean water and soap, and clean gloves worn for admission vaginal examination. Proportion of each birth practice successfully delivered	Hand hygiene compliance. Hand washing with soap and water or waterless alcohol-based hand rub^c^.	Hand hygiene compliance (% of times performed hand hygiene of all observed moments when required). Presumably, soap& water or handrub necessary^c^.
**Opportunity type**	Before contact with patient during delivery	Before aseptic procedures during labour and delivery (including vaginal examination)	Second stage of labour; unclear if before or after what type of contact	Before delivery	Before procedures in the labour and postnatal ward	Before delivery	Before examining patients	Before aseptic procedures during labour and delivery	Before or after delivery	Before gloving for delivery	5 types of WHO hand hygiene opportunities in the delivery suite e.g. before patient contact	Second stage of labour; unclear if before or after what type of contact	Before delivery	Before delivery	Before aseptic/clean procedures in the labour and emergency room
**Numerator**	1	7	Unclear	0	14	374	30^a^	75	15	300	142	Unclear	41^a^	80^a^	31^a^
**Denominator**	52	201	Unclear	18	37	1027	63	781	20	371	507	Unclear	388	235	116
**Compliance %**	1.9%^a^	4.0%	11.5%	0%^a^	37.8%^a^	36%	48%	9.6%	75.0%^a^	81.0%	28.0%^a^	< 20.0%^b^	10.6%^a^	34.0%	27.0%
**N individuals**	52 women	31 women	200 women	18 women	37 midwives	1277 deliveries	Unclear	104 birth attendants	20 women	371 women	Unclear	96 women	Unclear	235 deliveries	Unclear
**N facilities**	2	6	9	Unclear	2	15	63	10	1	101	1	2	1	35	1
**Cadre/** **intervention**	NA	NA	NA	NA	NA	NA	NA	NA	NA	NA	Before the intervention	NA	Before the intervention	NA	Doctors
**2nd Estimate**															
**Outcome definition**		As above		Hands were washed; unclear if with soap			As above			As above			As above	As above	As above
**Opportunity type**		Before vaginal examination		Before wound care for episiotomy and vaginal tears			Before putting on gloves			Before touching delivery area surfaces and equipment			Before vaginal examination	Before per-vaginal examination	Before aseptic/clean procedures in the labour and emergency room
**Numerator**		6		4			32^a^			289			5.3^a^	92^a^	4^a^
**Denominator**		121		4			63			371			405	242	18
**Compliance %**		5.0%		100%^a^			51.0%			78.0%			1.3%	38.0%	21.2%
**N individuals**		31 women		4 women			Unclear			371 women			Unclear	Unclear	Unclear
**N facilities**		6		Unclear			63			101			1	35	1
**Cadre/intervention**		NA		NA						NA			Before the intervention	NA	Nurses
**3rd Estimate**															
**Outcome definition**				Hands were washed; unclear if with soap											
**Opportunity type**				Cord care; unclear if before/after											
**Numerator**				9											
**Denominator**				22											
**Compliance %**				40.9%^a^											
**N individuals**				22 newborns											
**N facilities**				Unclear											
**Cadre/intervention**				NA											

^a^Estimates imputed by systematic review author

^b^Less than 20% was considered a level that is “not acceptable”. No exact estimate provided – estimated from Fig. 1 of Simbar et al.

^c^Followed WHO guidelines 2009 “WHO Guidelines on Hand Hygiene in Health Care: First Global Patient Safety Challenge Clean Care is Safer Care” or “Hand Hygiene Technical Reference Manual”

Another aspect of the definition is the type of hand hygiene opportunity (when hand hygiene should occur). The WHO hand hygiene guidelines refer to five key hand hygiene opportunities: before clean/clean procedures, after exposure to body fluids, before touching the patient, after touching the patient, after touching the patient’s surrounding. Studies did not always report what the type of contact (before vs. after; contact with intact skin i.e. “touching a patient” or non-intact/mucous membrane i.e. clean/aseptic procedures). Indeed, Changaee et al., and Simbar et al. were contacted for further information on their hand hygiene definition as it was unclear if it was before after the procedure/contact, but did not reply [29, 32]. Further enquires were also made to Yawson and Hesse, and Friday et al. on their definitions but with no reply [24, 32]. Another unclear area is what procedures during labour or delivery were captured. Studies that clearly outline this are Gon et al. and Buxton et al. [19, 25]

### Hand hygiene compliance estimates during labour and delivery

We extracted estimates that were clearly for aseptic procedures, and estimates for which this was not clear or where aseptic procedures were not the exclusive focus. Definitions across the studies were extremely heterogeneous and hence we did not combine their estimates; compliance estimates varied from 0 to 100%. Spector et al. reported a baseline compliance of 1.3% before vaginal examinations during admission and 10.6% before deliveries [30]. A follow up study of the same intervention by Delaney et al. reported compliance before delivery at 36% after 2 months of intervention measured by independent observers during non-intervention days [23]. Buxton et al. found an overall compliance of 4% before aseptic procedures during labour and delivery, and a compliance of 5% before vaginal examination [19]. Gon et al. reported overall compliance with hand rubbing/washing, glove use and avoiding recontamination in 9.6% of opportunities before aseptic procedures during labour and delivery [25]. Yawson and Hesse reported hand hygiene compliance before aseptic procedures across both the labour and emergency room (we assumed that the emergency room was primarily dedicated to pregnant women); among doctors, compliance was 27.0%, whereas among nurses it was 21.2% [32]. Phan et al. reported the baseline compliance to be 28% across five types of WHO hand hygiene opportunities (before patient contact, before aseptic task etc.) observed in the delivery suite [28]. Mannava et al. reported a compliance 81% before gloving for delivery [27]. Simbar et al. [29] and Changaee et al. [20] reported on compliance during second stage of labour, although it was unclear whether compliance was before or after interaction with the patient or which type of interaction i.e. aseptic procedure, touching the patient. Simbar et al. reported a compliance level below 20.0%, which they describe as unacceptable [29]. We could not interpret the estimate by Chanagaee et al. because the definition of compliance was ambiguous [20]. Asp et al. report a compliance of 1.9% before contact with patient during delivery or immediate postpartum; it is unclear if this includes aseptic procedures or not [18]. Hoogenboom et al. found that in 75.0% of deliveries, either before or after the delivery, at least one birth attendant present hand washed [26]. Danda et al. reported compliance before procedures (not clear what type) across the labour and postnatal wards – here, 37.8% of midwives washed their hands at least once [22]. Friday et al. measured compliance before examining patients in the labour ward (48%) and before putting on gloves (51%). However, the compliance represents the percentage of facilities, rather than opportunities or individuals, that comply [24]. Finally, Cronin et al. reported that the midwives scrub hands in none of the 18 deliveries they observed (currently this practice is not necessary before delivery); however, all used either water and soap, or Dettol to perform hand hygiene [21]. All the four observations of wound care in this study were preceded by hand washing (100%) but only 40.9% of the cord-care observations (not clear if before or after cord care).

Table 3 describes the estimates extracted related to “before aseptic procedures” opportunities, from the smallest to the largest, as well as whether we considered their sample size adequate, their definition sufficiently good and whether the authors provided isolated estimates specifically for opportunities before aseptic procedures during labour and delivery. Five studies presented better definitions and larger sample sizes, and were specific to aseptic procedures during labour and birth: Spector et al. [30]; Gon et al. [25]; Buxton et al. [19]; Tyagi et al. [31]; Delaney et al. [23].

**Table 3:** Selected compliance estimates summarised

% Compliance	Author	Type of opportunity	Sample size	Definition	Specific estimate ***before*** aseptic proc. during labour and delivery
**0**	Cronin [21]	Before delivery	Small	Suboptimal	No
**1.3**	Spector [30]	Before vaginal exam.	Adequate	Good	Yes
**1.9**	Asp [18]	Before contact	Adequate	Suboptimal	No
**4.0**	Buxton [19]	Before aseptic procedures	Adequate	Good	Yes
**5.0**	Buxton [19]	Before vaginal examination	Adequate	Good	Yes
**9.6**	Gon [25]	Before aseptic procedures	Adequate	Good	Yes
**10.6**	Spector [30]	Before delivery	Adequate	Good	Yes
**11.5**	Changaee [20]	II stage of labour	Adequate	Suboptimal	No
**< 20**	Simbar [29]	II stage of labour	Adequate	Suboptimal	No
**21.2**	Yawson [32]	Before aseptic (doct.)	Adequate	Satisfactory	Unclear^a^
**27.0**	Yawson [32]	Before aseptic (nurs.)	Adequate	Satisfactory	Unclear^a^
**28.0**	Phan [28]	All 5 types of opp.	Adequate	Satisfactory	No
**34.0**	Tyagi [31]	Before delivery	Adequate	Good	Yes
**36.0**	Delaney [23]	Before delivery (independent observers)	Adequate	Good	Yes
**37.8**	Danda [22]	Before procedures	Small	Suboptimal	No
**38.0**	Tyagi [31]	Before vaginal examination	Adequate	Good	Yes
**40.9**	Cronin [21]	During cord care	Small	Suboptimal	Yes
**48.0**	Friday [24]	Before examining patients	Unclear	Suboptimal	Unclear
**51.0**	Friday [24]	Before putting on gloves	Unclear	Suboptimal	Unclear
**75.0**	Hoogenboom [26]	During delivery	Small	Suboptimal	No
**78.0**	Mannava [27]	Before touching any delivery areas or surface	Adequate	Satisfactory	Yes
**81.0**	Mannava [27]	Before gloving for delivery	Adequate	Satisfactory	Yes
**100**	Cronin [21]	Before wound care	Small	Satisfactory	Yes

^a^Emergency room may not only cater for labouring women

### Technique and duration of hand hygiene, and avoiding recontamination

Only three studies [21, 25, 32] reported on aspects of hand hygiene quality such as technique and duration. Cronin et al. reported qualitatively that hand washings were generally not timed (not within the expected duration). Yawson and Hesse reported that on the labour ward, 50% or more of staff used soap and running water for hand washing, and dried hands with clean single use towels. Less than 50% washed hands for 40–60 s, or cleaned hands with alcohol handrub, or performed the appropriate handwashing technique [32]. Gon et al. reported the level of adequate rubbing/washing technique at 30.7% [25] defined as one of the hand gesture required by the WHO technical reference manual [35] i.e. “right palm over left dorsum with interlaced fingers and vice versa”; adequate duration was at 14.6% defined as ≥10 s based on the local guidelines for infection prevention [25].

Cronin et al. discuss qualitatively the concept of avoiding hand or glove recontamination before a procedure. This is a quote from their article.

“*frequent breaks in technique included … the midwife’s gloved hands touching the patient’s bed, leg, abdomen, and perineal pad before the delivery*.” [37].

Gon et al. defined recontamination of hands or gloves as any touch on potentially contaminated surfaces within the workflow after glove donning or hand rubbing/washing when preparing for a an aseptic procedure e.g. touching an unclean delivery surface, unclean hand-drying material, the woman and newborn outside the defined patient zone, the woman’s bed, trolley, unclean objects used during hand hygiene, and other unclean surfaces, unless classified as outside the workflow and provide an exhaustive list of these actions and that of patient zone within which touching surfaces is allowed [25]. They report that birth attendants risked recontaminating their hands or gloves in 45.3% of the opportunities when rubbing/washing or glove donning occurred [25].

Buxton et al. reported avoiding recontamination as part of hand hygiene compliance in the most hygienic category but did not specify the definition of what behaviours are included in recontamination [19].

### Interventions, effect size for hand hygiene determinants and ICC

Three studies report interventions aimed at increasing hand hygiene compliance. Two studies relied on a pre-post intervention design, without randomization or control wards; one only reported the intervention period without baseline. The three studies reported on interventions including several components – two of these studies discuss the same intervention. Phan et al. [28] tested an educational program on hand hygiene provided to healthcare workers over two 3 h sessions. The educational model used experiential learning and incorporated novel techniques of learning that allowed for consideration of past hand hygiene experiences. Fifty two out of 53 healthcare staff in the delivery suite participated in the intervention. The intervention improved hand hygiene overall in the selected wards, but the effect was largest in the delivery suite increasing from 28 to 61.8% across all five types of WHO hand hygiene opportunities [28]. The improvement was sustained over a period of 6 months of post intervention follow-up. Given the nature of the intervention, we assumed that participants were not blinded to the aim of the intervention.

Spector et al. tested a four-components childbirth safety program based on the WHO Safe Childbirth Checklist [30]. After the intervention, hand hygiene compliance increased respectively from 1.3 to 97.8% before vaginal examination during admission and from 10.6 to 99.5% before delivery. The checklist included prompts on elements of hand hygiene; therefore, the healthcare workers were not blinded to the aim of the intervention. Delaney et al. [23] also describes the introduction of the WHO’s Safe Childbirth Checklist. This was part of a large randomised control trial, but the article included here focuses on the 60 facilities that received the intervention. There is no control or baseline group for comparing hand hygiene without the intervention. The main comparison is between the first month of intervention and the latter 7–8 months carried out by the same peer-coaches who run the intervention – compliance before delivery was respectively 76% and 94%. The independent assessment of hand hygiene described above showed compliance at 36% between 2 and 5 months of the intervention period. Given the presence of peer coaching, participants were not blinded to the aim of the intervention.

A few studies looked quantitatively at the association between potential determinants and hand hygiene compliance (measured via observation or other objective method) – but none of these were individual level determinants except for cadre. These appear to be all unadjusted associations. Mannava et al. reported that ﻿hand hygiene compliance before touching any delivery surfaces was lower in tertiary hospitals at 71%, vs 83% for first-level referral hospitals (*p*-value < 0.001), and higher ﻿in hospitals where all delivery rooms had soap and a sink with water compared to hospitals where needed supplies was not available in all rooms (50% vs 39%, *p*-value = 0.29) [27]. Buxton et al. tested the association between hand hygiene compliance and cadre, national state, and facility type - these were not found to be associated; they do find an association with shift – with the morning shift having higher compliance compared to the afternoon (*p*-value = 0.0034) and night (*p*-value = 0.008) [19]. Tyagi et al. described hand hygiene compliance by facility type, reporting a compliance of 100% in private facilities compared to 27% in public facilities (*p*-value = 0.011) [31]. They do not find an association with facility level and facility load [31]. Gon et al. report that hand hygiene compliance did not vary much by observer or by shift, indeed the confidence intervals overlapped across the of these categories [25].

With regards to the ICC, we present here the results we gathered from studies with the larger sample size and clearer definitions, involving more than two facilities, and where authors replied to our request. Estimates of rho in Buxton et al. [19] and Gon et al. [25], are both closer to 0 than 1 indicating that variance within facilities appear higher than between facilities (Table 4).

**Table 4:** ICC results

	Buxton et al. [19]	Gon et al. [25]
**Outcome is hand hygiene during:**	Before aseptic procedures during labour/delivery	Before aseptic procedures during labour/delivery
**Facilities**	6	10
**Numerator**	7	75
**Denominator**	201	781
**Rho**	< 0.0001	0.13

ICC for the variation between and within individuals is also provided by Gon et al. and reports higher variance within than between individuals [25].

## Discussion

We performed a systematic review of published studies reporting estimates of birth attendants’ hand hygiene compliance conducted in healthcare facilities in LMICs. We found fifteen studies that met our inclusion criteria. Hand hygiene compliance estimates were extremely diverse, ranging from 0 to 100%; the heterogeneity in definitions of hand hygiene did not allow us to combine or compare these meaningfully. Four studies (Cronin et al., Hoogenboom et al., Friday et al., and Mannava et al.) reported higher compliance. Except for Mannava et al., these with higher compliance also had a very small or unclear sample, and used an individual level or group level definition for the denominator rather than the number of patient-attendant interactions (hand hygiene opportunities) as recommended by the WHO hand hygiene guidelines [21, 22, 26]. The studies [19, 23, 25, 28, 30–32] with larger sample sizes and clearer definitions suggest compliance to **hand hygiene before aseptic procedures** to be low, between 1.3 and 38.0%. We have three estimates for hand hygiene before vaginal examination which spans between 1.3% [30] and 38% [31]; and we have five estimates for hand hygiene before labour/delivery-related procedures spans between 4 and 36% [23]. Overall, the quality of the included studies was particularly compromised by poorly described sampling methods and definitions.

The studies included were published in the last 18 years and spanned 14 countries between Sub-Saharan Africa, South East Asia and the Middle East. Four studies only included one facility, limiting their generalizability. The supplies of key hand hygiene infrastructure were poorly described, except in four studies. The quality of the studies included was generally poor with a high risk of bias with a few exceptions. The weakest aspect of the studies was their description of the sampling strategy, as most studies did not describe how the unit of observation was sampled (whether women, healthcare workers or specific procedures). Also, the reported definitions of hand hygiene were often incomplete. For most studies it was unclear whether the use of soap was a necessary condition to achieve hand washing compliance. In addition, the type of hand hygiene opportunity was often poorly described i.e. before or after the interaction with the patient; aseptic procedures vs. contact with the patient intact skin. Finally, in four studies the denominator did not rely on patient-worker interactions but on the overall performance of an individual or a group, or on the number facilities were hand hygiene was observed. This finding, of poor methods in conducting and reporting of observational studies on hand hygiene and more broadly of healthcare workers, was reported elsewhere [6, 36].

Beyond the basic aspects of quality required for any observational study and described by the STROBE guidelines [15], future studies focusing on hand hygiene during labour and delivery should design and report the following more clearly:

- what sampling strategy was used to observe either workers, women, or patient-worker interactions; and how facilities visits were scheduled;
- the methods used to ensure the quality of data collection in the study e.g. data monitoring
- the inter-observer agreement where multiple observers are employed;
- the definition of hand hygiene using the WHO hand hygiene guidelines [37] (i.e. soap necessary for hand washing; which type of hand hygiene opportunity e.g. before vs. after, touching intact skin vs. aseptic procedure; denominator based on patient-worker interactions rather than individual or group level performance; types of procedures involved in the aseptic procedure; sequence of actions required to comply to hand hygiene);

Our findings of low birth attendants’ hand hygiene compliance are consistent with other systematic reviews or multi-country studies in LMICs of hand hygiene among healthcare workers more generally, which report compliance estimates ranging from 22 to 35% during non-intervention periods [38, 39]. Similarly to these studies, our estimates point to a slight lower compliance in LMICs compared to high-income settings. With approximately 140 million women delivering worldwide, most of which are in LMICs and at least half of which occur in healthcare facilities where quality of care is suboptimal, these low estimates of hand hygiene compliance during labour/delivery are worrisome [5, 40, 41]. If correct, these estimates pose a substantial risk to infection prevention during birth in LMICs where both mothers and newborns are still largely affected by infection [1, 2, 42].

None of the included studies specifically investigated the wide range of individual determinants of hand hygiene compliance – except for cadre examined in one study. Four however report compliance estimates by study or facility characteristics. Three studies [30, 32] investigated the effect of two different interventions on hand hygiene, a checklist on quality of care at birth and an education program. Both were successful in increasing substantially the hand hygiene compliance during labour/delivery. Given the nature of their study design – pre-post intervention without a control ward, or without baseline, and with study participants who are no blinded – these interventions tell us more about the feasibility of these interventions in these specific contexts compared to anything conclusive about their scope for improving hand hygiene more widely in LMICs. With regards to ICC, from 2 studies we find that variation is greater within than between facilities.

Our systematic review covered four separate databases, has a clearly reported search strategy adapted from previous systematic reviews on the topic, did not pose any restrictions based on language, and used independent double full text screening and article extraction. A potential weakness is that our search might have missed articles which included hand hygiene in the broader framework of quality of care during birth or infection prevention and control and which did not mention hand hygiene in their title or abstract. We did not assess publication bias, but this would be more of an issue for intervention studies that found negative results for example than for observational studies reporting on compliance estimates. Finally, the set of health care facilities included in this systematic review is unlikely to represent health care facilities across LMICs. Without random sampling from the reference population of health care facilities, estimates of hand hygiene may be subject to selection bias stemming from researchers non-random decisions about which facilities to study. For example, researchers may be more likely to sample from higher volume facilities where deliveries are frequent than to sample from lower volume facilities. Studies suggest that higher volume facilities are better equipped for attending deliveries, but they maybe more prone to crowding which in turn makes hand hygiene more challenging [43]. Only Gon et al., Mannava et al., Tyagi et al. [25, 27, 31] can be regarded representative of the reference population which they targeted, respectively: high-volume labour wards in Zanzibar, hospitals implementing EENC in the countries included from South East Asia, hospitals with a newborn unit in Andhra Pradesh and Telengana regions of India who did not receive a quality improvement intervention. It is hard to make this inference for Friday et al. because of their group level definition of hand hygiene. [24]

## Conclusions

In conclusion, we found fifteen articles reporting the hand hygiene compliance of healthcare workers during labour and delivery in LMICs. Compliance including before aseptic procedures opportunities for studies with larger sample sizes and clear definitions was low, ranging between 1 and 38%. This is an opportunity for infection prevention reduction during birth in LMICs facilities since effective interventions in this area are likely to reduce infection rate among mothers and newborns. We also found that the quality of many studies was suboptimal. In particular, future studies of hand hygiene compliance during the labour ward should be designed with better sampling frame, assess inter-observer agreement, use measures to improve quality of data collection and report their hand hygiene definitions clearly.

## Supplementary Information

**Additional file 1.** “Systematic review search strategy” – it includes the search strategy for each database used in our review.

**Additional file 2.** “PRISMA 2009 Checklist” – It includes the details of our manuscript against the PRISMA checklist.

## Data Availability

All data generated or analysed during this study are included in this published article.

## Abbreviations

CONSORT: Consolidated Standards of Reporting Trials
HICs: High Income Countries
ICC: Intra-cluster correlation LMICs
PRISMA: Preferred Reporting Items for Systematic Reviews and Meta-Analyses
STROBE: Strengthening the Reporting of Observational studies in Epidemiology
WHO: World Health Organisation
