# Supplementary material for: Birth attendants’ hand hygiene compliance in healthcare facilities in low and middle-income countries: a systematic review
Source: BMC Health Serv Res. 2020 Dec 3;20:1116. doi: 10.1186/s12913-020-05925-9 (PMC7713338; doi:10.1186/s12913-020-05925-9)
Supplement: Supplementary file 1 — Additional file 1. “Systematic review search strategy” – it includes the search strategy for each database used in our review. [file 12913_2020_5925_MOESM1_ESM.docx]

### Additional File 1 - Systematic review search strategy

**EMBASE**

1. Handwashing/
2. (hand antisepsis or handwash$ or hand wash$ or hand disinfection or hand hygiene or surgical scrub$).tw.
3. exp Hand/
4. exp Sterilization/
5. 1 or 2
6. 3 and 4
7. 5 or 6
8. exp maternity ward/
9. (maternit* or gynaecology* or gynecolog* or labour or labor or birth* or deliver* or obstetric* or childbirth* or intrapartum).mp. [mp=title, abstract, heading word, drug trade name, original title, device manufacturer, drug manufacturer, device trade name, keyword]
10. 8 or 9
11. 7 and 10
12. Limit to Low and Middle Income Countries (LMICs – see full list below)
13. 11 and 12

**MEDLINE**

1. Handwashing/
2. (hand antisepsis or handwash$ or hand wash$ or hand disinfection or hand hygiene or surgical scrub$).tw.
3. exp Hand/
4. exp Sterilization/
5. 1 or 2
6. 3 and 4
7. 5 or 6
8. exp Hospitals, Maternity/
9. (maternity* or gynaecolog* or gynecolog* or labour or labor or deliver* or birth* or obstetric* or childbirth* or intrapartum).mp. [mp=title, abstract, heading word, drug trade name, original title, device manufacturer, drug manufacturer, device trade name, keyword]
10. 8 or 9
11. 7 and 10
12. Limit to LMICs (see full list below)
13. 11 and 12

**CINHAL Plus**

1. (MH* "Handwashing+")
2. (hand antisepsis or handwash* or hand

wash* or hand disinfection or hand hygiene

or surgical scrub*)

1. 1 or 2
2. Hand*
3. Sterilization*
4. 4 and 5
5. 3 or 6
6. (MH "Delivery Rooms+")
7. Maternity* or gynaecolog* or gynecolog* or labour or labor or deliver* or birth* or obstetric* or childbirth or intrapartum
8. 8 or 9
9. 10 and 7
10. **Limiters** – Mexico and South America, Asia, Africa, Middle East

**WHO regional databases**

(hand antiseps* handwash* OR hand hygiene OR hand wash* OR hand disinfection OR surgical scrub OR hand sterilization) AND (Maternit* or gynaecolog* or gynecolog* or labour or labor or deliver* or birth* or obstetric* or childbirth* or intrapart*)

**LMICs country search strategy (developed by the London School of Hygiene and Tropical Medicine librarian)**

**EMBASE**

1. developing country/
2. low income country/
3. middle income country/
4. ((developing or less* developed or under developed or underdeveloped or middle income or low* income or underserved or under served or deprived or poor*) adj (economy or economies)).ti,ab.
5. ((developing or less* developed or under developed or underdeveloped or middle income or low* income or underserved or under served or deprived or poor*) adj (countr* or nation? or population? or world)).ti,ab.
6. (low* adj (gdp or gnp or gross domestic or gross national)).ti,ab.
7. (low adj3 middle adj3 countr*).ti,ab.
8. (lmic or lmics or third world or lami countr*).ti,ab.
9. transitional countr*.ti,ab.
10. global south.ti,ab.
11. "Africa south of the Sahara"/
12. ("africa south of the sahara" or sub-saharan africa or central africa or eastern africa or southern africa or western africa).ti,ab.
13. Botswana/
14. (Botswana or Bechuanaland or Kalahari).ti,ab.
15. Equatorial Guinea/
16. (Equatorial Guinea or Spanish Guinea).ti,ab.
17. Gabon/
18. (Gabon or Gabonese Republic).ti,ab.
19. Mauritius/
20. (Mauritius or Agalega Islands).ti,ab.
21. Namibia/
22. Namibia.ti,ab.
23. South Africa/
24. South Africa.ti,ab.
25. Angola/
26. angola.ti,ab.
27. Cameroon/
28. Cameroon.ti,ab.
29. Cape Verde/
30. (Cape Verde or Cabo Verde).ti,ab.
31. Congo/
32. (congo not ((democratic republic adj3 congo) or congo red or crimean-congo)).ti,ab.
33. Cote d'Ivoire/
34. (Cote d'Ivoire or Ivory Coast).ti,ab.
35. Ghana/
36. (Ghana or Gold Coast).ti,ab.
37. Kenya/
38. kenya.mp.
39. Lesotho/
40. (Lesotho or Basutoland).ti,ab.
41. Mauritania/
42. Mauritania.ti,ab.
43. Nigeria/
44. Nigeria.ti,ab.
45. "Sao Tome and Principe"/
46. (sao tome adj2 principe).ti,ab.
47. Sudan/
48. (Sudan not south sudan).ti,ab.
49. Swaziland/
50. Swaziland.ti,ab.
51. Zambia/
52. (Zambia or Northern Rhodesia).ti,ab.
53. Benin/
54. (Benin or Dahomey).ti,ab.
55. Burkina Faso/
56. (Burkina Faso or Burkina Fasso or Upper Volta).ti,ab.
57. Burundi/
58. Burundi.ti,ab.
59. Central African Republic/
60. (Central African Republic or Ubangi-Shari).ti,ab.
61. Chad/
62. Chad.ti,ab.
63. Comoros/
64. (Comoros or Comoro Islands or Mayotte or Iles Comores).ti,ab.
65. "Democratic Republic Congo"/
66. ((democratic republic adj2 congo) or belgian congo or zaire).ti,ab.
67. Eritrea/
68. Eritrea.ti,ab.
69. Ethiopia/
70. Ethiopia.ti,ab.
71. Gambia/
72. Gambia.ti,ab.
73. Guinea/
74. (Guinea not (New Guinea or Guinea Pig* or Guinea Fowl)).ti,ab.
75. Guinea-Bissau/
76. (Guinea-Bissau or Portuguese Guinea).ti,ab.
77. Liberia/
78. Liberia.ti,ab.
79. Madagascar/
80. (Madagascar or Malagasy Republic).ti,ab.
81. Malawi/
82. (Malawi or Nyasaland).ti,ab.
83. Mali/
84. Mali.ti,ab.
85. Mozambique/
86. (Mozambique or Mocambique or Portuguese East Africa).ti,ab.
87. Niger/
88. (Niger not (Aspergillus or Peptococcus or Schizothorax or Cruciferae or Gobius or Lasius or Agelastes or Melanosuchus or radish or Parastromateus or Orius or Apergillus or Parastromateus or Stomoxys)).ti,ab.
89. Rwanda/
90. (Rwanda or Ruanda).ti,ab.
91. Senegal/
92. senegal.ti,ab.
93. Sierra Leone/
94. Sierra Leone.mp.
95. exp Somalia/
96. Somalia.ti,ab.
97. South Sudan/
98. south sudan.ti,ab.
99. Tanzania/
100. (Tanzania or Tanganyika or Zanzibar).ti,ab.
101. Togo/
102. (Togo or Togolese Republic).ti,ab.
103. Uganda/
104. Uganda.ti,ab.
105. Zimbabwe/
106. (Zimbabwe or Rhodesia).ti,ab.
107. Maldives/
108. Maldives.ti,ab.
109. Algeria/
110. Algeria.ti,ab.
111. Iran/
112. Iran.ti,ab.
113. exp Iraq/
114. Iraq.ti,ab.
115. Jordan/
116. Jordan.ti,ab.
117. Lebanon/
118. Lebanon.ti,ab.
119. Libyan Arab Jamahiriya/
120. Libya.ti,ab.
121. Argentina/
122. Argentina.ti,ab.
123. Belize/
124. Belize.ti,ab.
125. exp Brazil/
126. Brazil.ti,ab.
127. Colombia/
128. Colombia.ti,ab.
129. Costa Rica/
130. Costa Rica.ti,ab.
131. Cuba/
132. Cuba.ti,ab.
133. Dominica/
134. Dominica.ti,ab.
135. Dominican Republic/
136. Dominican Republic.ti,ab.
137. Ecuador/
138. Ecuador.ti,ab.
139. Grenada/
140. Grenada.ti,ab.
141. Guyana/
142. Guyana.mp.
143. Jamaica/
144. Jamaica.ti,ab.
145. Mexico/
146. Mexico.ti,ab.
147. exp Panama/
148. Panama.ti,ab.
149. Paraguay/
150. Paraguay.mp.
151. Peru/
152. Peru.ti,ab.
153. Saint Lucia/
154. (St Lucia or Saint Lucia).ti,ab.
155. "Saint Vincent and the Grenadines"/
156. Grenadines.ti,ab.
157. Suriname/
158. Suriname.ti,ab.
159. Venezuela/
160. Venezuela.ti,ab.
161. Albania/
162. Albania.ti,ab.
163. Azerbaijan/
164. Azerbaijan.ti,ab.
165. Belarus/
166. (belarus or byelarus or belorussia).ti,ab.
167. exp "Bosnia and Herzegovina"/
168. (bosnia or herzegovina).ti,ab.
169. Bulgaria/
170. Bulgaria.ti,ab.
171. Croatia/
172. croatia.ti,ab.
173. Kazakhstan/
174. (Kazakhstan or kazakh).ti,ab.
175. "Macedonia (Republic)"/
176. Macedonia.ti,ab.
177. "Montenegro (republic)"/
178. Montenegro.ti,ab.
179. Romania/
180. Romania.ti,ab.
181. exp Russian Federation/
182. USSR/
183. (Russia or Russian Federation or USSR or Union of Soviet Socialist Republics or Soviet Union).mp.
184. exp Serbia/
185. serbia.ti,ab.
186. "Turkey (republic)"/
187. turkey.ti,ab. not animal/
188. Turkmenistan/
189. Turkmenistan.ti,ab.
190. Yugoslavia/
191. yugoslavia.ti,ab.
192. exp Samoan Islands/
193. american samoa.ti,ab.
194. exp China/
195. china.ti,ab.
196. Fiji/
197. fiji.ti,ab.
198. Malaysia/
199. malaysia.ti,ab.
200. Marshall Islands/
201. marshall islands.ti,ab.
202. Nauru/
203. nauru.ti,ab.
204. ("independent state of samoa" or (samoa not american samoa) or western samoa or navigator islands or samoan islands).ti,ab.
205. Thailand/
206. Thailand.ti,ab.
207. Tonga/
208. tonga.ti,ab.
209. Tuvalu/
210. Tuvalu.ti,ab.
211. Bangladesh/
212. Bangladesh.ti,ab.
213. Bhutan/
214. Bhutan.ti,ab.
215. exp India/
216. India.ti,ab.
217. exp Pakistan/
218. Pakistan.ti,ab.
219. Sri Lanka/
220. Sri Lanka.ti,ab.
221. Djibouti/
222. (Djibouti or French Somaliland).ti,ab.
223. Egypt/
224. Egypt.ti,ab.
225. Jordan/
226. Jordan.ti,ab.
227. Morocco/
228. Morocco.ti,ab.
229. Syrian Arab Republic/
230. (Syria or Syrian Arab Republic).ti,ab.
231. Tunisia/
232. tunisia.mp.
233. Palestine/
234. Gaza.ti,ab.
235. Yemen/
236. Yemen.ti,ab.
237. Bolivia/
238. Bolivia.ti,ab.
239. El Salvador/
240. El Salvador.ti,ab.
241. Guatemala/
242. Guatemala.ti,ab.
243. Honduras/
244. Honduras.ti,ab.
245. Nicaragua/
246. Nicaragua.ti,ab.
247. Armenia/
248. Armenia.ti,ab.
249. "Georgia (Republic)"/
250. Kosovo/
251. Kosovo.ti,ab.
252. Kyrgyzstan/
253. (kyrgyzstan or kyrgyz republic or kirghizia or kirghiz).ti,ab.
254. Moldova/
255. Moldova.ti,ab.
256. Tajikistan/
257. tajikistan.ti,ab.
258. exp Ukraine/
259. Ukraine.ti,ab.
260. Uzbekistan/
261. Uzbekistan.ti,ab.
262. Cambodia/
263. cambodia.ti,ab.
264. exp Indonesia/
265. indonesia.ti,ab.
266. Kiribati/
267. Kiribati.ti,ab.
268. Laos/
269. (laos or (lao adj1 democratic republic)).ti,ab.
270. "Marshall Islands"/
271. "Federated States of Micronesia"/
272. (marshall island* or caroline island* or ellice island* or gilbert island* or johnston island* or mariana island* or micronesia or pacific island*).ti,ab.
273. Mongolia/
274. mongolia.ti,ab.
275. Myanmar/
276. (myanmar or burma).ti,ab.
277. Papua New Guinea/
278. Papua New Guinea.ti,ab.
279. Philippines/
280. Philippines.ti,ab.
281. Timor-Leste/
282. Timor-Leste.ti,ab.
283. Vanuatu/
284. Vanuatu.ti,ab.
285. Viet Nam/
286. (Viet Nam or vietnam).ti,ab.
287. Afghanistan/
288. Afghanistan.ti,ab.
289. Nepal/
290. Nepal.ti,ab.
291. Haiti/
292. Haiti.ti,ab.
293. "North Korea"/
294. (north korea or (democratic people* republic adj2 korea)).ti,ab.
295. or/12-305 [ALL LMICs]
296. 11 and 306

**MEDLINE**

1. ((developing or less* developed or under developed or underdeveloped or middle income or low* income or underserved or under served or deprived or poor*) adj (economy or economies)).ti,ab.
2. ((developing or less* developed or under developed or underdeveloped or middle income or low* income or underserved or under served or deprived or poor*) adj (countr* or nation? or population? or world)).ti,ab.
3. (low* adj (gdp or gnp or gross domestic or gross national)).ti,ab.
4. (low adj3 middle adj3 countr*).ti,ab.
5. (lmic or lmics or third world or lami countr*).ti,ab.
6. transitional countr*.ti,ab.
7. global south.ti,ab.
8. Developing Countries/
9. "africa south of the sahara"/ or africa, central/ or africa, eastern/ or africa, southern/ or africa, western/
10. ("africa south of the sahara" or sub-saharan africa or central africa or eastern africa or southern africa or western africa).ti,ab.
11. "Democratic People's Republic of Korea"/
12. (north korea or (democratic people* republic adj2 korea)).ti,ab.
13. Cambodia/
14. cambodia.ti,ab.
15. Indonesia/
16. indonesia.ti,ab.
17. Micronesia/
18. Kiribati.ti,ab.
19. Laos/
20. (laos or (lao adj1 democratic republic)).ti,ab.
21. (marshall island* or caroline island* or ellice island* or gilbert island* or johnston island* or mariana island* or micronesia or pacific island*).ti,ab.
22. Mongolia/
23. mongolia.ti,ab.
24. Myanmar/
25. (myanmar or burma).ti,ab.
26. Papua New Guinea/
27. Papua New Guinea.ti,ab.
28. Philippines/
29. Philippines.ti,ab.
30. Timor-Leste/
31. Timor-Leste.ti,ab.
32. Vanuatu/
33. Vanuatu.ti,ab.
34. Vietnam/
35. (Viet Nam or Vietnam).ti,ab.
36. American Samoa/
37. american samoa.ti,ab.
38. exp China/
39. china.ti,ab.
40. Fiji/
41. fiji.ti,ab.
42. Malaysia/
43. malaysia.ti,ab.
44. marshall islands.ti,ab.
45. nauru.ti,ab.
46. samoa/
47. "independent state of samoa"/
48. ("independent state of samoa" or (samoa not american samoa) or western samoa or navigator islands or samoan islands).ti,ab.
49. Thailand/
50. Thailand.ti,ab.
51. Tonga/
52. tonga.ti,ab.
53. Tuvalu.ti,ab.
54. Armenia/
55. Armenia.ti,ab.
56. "Georgia (Republic)"/
57. Kosovo/
58. Kosovo.ti,ab.
59. Kyrgyzstan/
60. (kyrgyzstan or kyrgyz republic or kirghizia or kirghiz).ti,ab.
61. Moldova/
62. Moldova.ti,ab.
63. Tajikistan/
64. tajikistan.ti,ab.
65. Ukraine/
66. Ukraine.ti,ab.
67. Uzbekistan/
68. Uzbekistan.ti,ab.
69. Albania/
70. Albania.ti,ab.
71. Azerbaijan/
72. Azerbaijan.ti,ab.
73. "Republic of Belarus"/
74. (belarus or byelarus or belorussia).ti,ab.
75. Bosnia-Herzegovina/
76. (bosnia or herzegovina).ti,ab.
77. Bulgaria/
78. Bulgaria.ti,ab.
79. Croatia/
80. croatia.ti,ab.
81. Kazakhstan/
82. (Kazakhstan or kazakh).ti,ab.
83. "Macedonia (Republic)"/
84. Macedonia.ti,ab.
85. Montenegro/
86. Montenegro.ti,ab.
87. Romania/
88. Romania.ti,ab.
89. exp Russia/
90. USSR/
91. (Russia or Russian Federation or USSR or Union of Soviet Socialist Republics or Soviet Union).mp.
92. Serbia/
93. serbia.ti,ab.
94. Turkey/
95. turkey.ti,ab. not animal/
96. Turkmenistan/
97. Turkmenistan.ti,ab.
98. Yugoslavia/
99. yugoslavia.ti,ab.
100. Haiti/
101. Haiti.ti,ab.
102. Bolivia/
103. Bolivia.ti,ab.
104. El Salvador/
105. El Salvador.ti,ab.
106. Guatemala/
107. Guatemala.ti,ab.
108. Honduras/
109. Honduras.ti,ab.
110. Nicaragua/
111. Nicaragua.ti,ab.
112. Argentina/
113. Argentina.ti,ab.
114. Belize/
115. Belize.ti,ab.
116. Brazil/
117. Brazil.ti,ab.
118. Colombia/
119. Colombia.ti,ab.
120. Costa Rica/
121. Costa Rica.ti,ab.
122. Cuba/
123. Cuba.ti,ab.
124. Dominica/
125. Dominica.ti,ab.
126. Dominican Republic/
127. Dominican Republic.ti,ab.
128. Ecuador/
129. Ecuador.ti,ab.
130. Grenada/
131. Grenada.ti,ab.
132. Guyana/
133. Guyana.mp.
134. Jamaica/
135. Jamaica.ti,ab.
136. Mexico/
137. Mexico.ti,ab.
138. exp Panama/
139. Panama.ti,ab.
140. Paraguay/
141. Paraguay.mp.
142. Peru/
143. Peru.ti,ab.
144. Saint Lucia/
145. (St Lucia or Saint Lucia).ti,ab.
146. "Saint Vincent and the Grenadines"/
147. Grenadines.ti,ab.
148. Suriname/
149. Suriname.ti,ab.
150. Venezuela/
151. Venezuela.ti,ab.
152. Djibouti/
153. (Djibouti or French Somaliland).ti,ab.
154. Egypt/
155. Egypt.ti,ab.
156. Jordan/
157. Jordan.ti,ab.
158. Morocco/
159. Morocco.ti,ab.
160. Syria/
161. (Syria or Syrian Arab Republic).ti,ab.
162. Tunisia/
163. tunisia.mp.
164. Gaza.ti,ab.
165. Yemen/
166. Yemen.ti,ab.
167. Algeria/
168. Algeria.ti,ab.
169. Iran/
170. Iran.ti,ab.
171. Iraq/
172. Iraq.ti,ab.
173. Jordan/
174. Jordan.ti,ab.
175. Lebanon/
176. Lebanon.ti,ab.
177. Libya/
178. Libya.ti,ab.
179. Afghanistan/
180. Afghanistan.ti,ab.
181. Nepal/
182. Nepal.ti,ab.
183. Bangladesh/
184. Bangladesh.ti,ab.
185. Bhutan/
186. Bhutan.ti,ab.
187. exp India/
188. India.ti,ab.
189. Pakistan/
190. Pakistan.ti,ab.
191. Sri Lanka/
192. Sri Lanka.ti,ab.
193. Indian Ocean Islands/
194. Maldives.ti,ab.
195. Benin/
196. (Benin or Dahomey).ti,ab.
197. Burkina Faso/
198. (Burkina Faso or Burkina Fasso or Upper Volta).ti,ab.
199. Burundi/
200. Burundi.ti,ab.
201. Central African Republic/
202. (Central African Republic or Ubangi-Shari).ti,ab.
203. Chad/
204. Chad.ti,ab.
205. Comoros/
206. (Comoros or Comoro Islands or Mayotte or Iles Comores).ti,ab.
207. "Democratic Republic of the Congo"/
208. ((democratic republic adj2 congo) or belgian congo or zaire).ti,ab.
209. Eritrea/
210. Eritrea.ti,ab.
211. Ethiopia/
212. Ethiopia.ti,ab.
213. Gambia/
214. Gambia.ti,ab.
215. Guinea/
216. (Guinea not (New Guinea or Guinea Pig* or Guinea Fowl)).ti,ab.
217. Guinea-Bissau/
218. (Guinea-Bissau or Portuguese Guinea).ti,ab.
219. Liberia/
220. Liberia.ti,ab.
221. Madagascar/
222. (Madagascar or Malagasy Republic).ti,ab.
223. Malawi/
224. (Malawi or Nyasaland).ti,ab.
225. Mali/
226. Mali.ti,ab.
227. Mozambique/
228. (Mozambique or Mocambique or Portuguese East Africa).ti,ab.
229. Niger/
230. (Niger not (Aspergillus or Peptococcus or Schizothorax or Cruciferae or Gobius or Lasius or Agelastes or Melanosuchus or radish or Parastromateus or Orius or Apergillus or Parastromateus or Stomoxys)).ti,ab.
231. Rwanda/
232. (Rwanda or Ruanda).ti,ab.
233. Senegal/
234. senegal.ti,ab.
235. Sierra Leone/
236. Sierra Leone.mp.
237. Somalia/
238. Somalia.ti,ab.
239. South Sudan/
240. south sudan.ti,ab.
241. Tanzania/
242. (Tanzania or Tanganyika or Zanzibar).ti,ab.
243. Togo/
244. (Togo or Togolese Republic).ti,ab.
245. Uganda/
246. Uganda.ti,ab.
247. Zimbabwe/
248. (Zimbabwe or Rhodesia).ti,ab.
249. Angola/
250. angola.ti,ab.
251. Cameroon/
252. Cameroon.ti,ab.
253. Cape Verde/
254. (Cape Verde or Cabo Verde).ti,ab.
255. Congo/
256. (congo not ((democratic republic adj3 congo) or congo red or crimean-congo)).ti,ab.
257. Cote d'Ivoire/
258. (Cote d'Ivoire or Ivory Coast).ti,ab.
259. Ghana/
260. (Ghana or Gold Coast).ti,ab.
261. Kenya/
262. kenya.mp.
263. Lesotho/
264. (Lesotho or Basutoland).ti,ab.
265. Mauritania/
266. Mauritania.ti,ab.
267. Nigeria/
268. Nigeria.ti,ab.
269. Atlantic Islands/
270. (sao tome adj2 principe).ti,ab.
271. Sudan/
272. (Sudan not south sudan).ti,ab.
273. Swaziland/
274. Swaziland.ti,ab.
275. Zambia/
276. (Zambia or Northern Rhodesia).ti,ab.
277. Botswana/
278. (Botswana or Bechuanaland or Kalahari).ti,ab.
279. Equatorial Guinea/
280. (Equatorial Guinea or Spanish Guinea).ti,ab.
281. Gabon/
282. (Gabon or Gabonese Republic).ti,ab.
283. Mauritius/
284. (Mauritius or Agalega Islands).ti,ab.
285. Namibia/
286. Namibia.ti,ab.
287. South Africa/
288. South Africa.ti,ab.
289. or/1-288 [ALL LMIC]
